# Supplementary material for: Angiotensin II Promotes Osteocyte RANKL Expression via AT1R Activation
Source: Biomedicines. 2025 Feb 10;13(2):426. doi: 10.3390/biomedicines13020426 (PMC11853621; doi:10.3390/biomedicines13020426)
Supplement: Supplementary file 1 [file biomedicines-13-00426-s001.zip › biomedicines-3415696-supplementary.pdf]

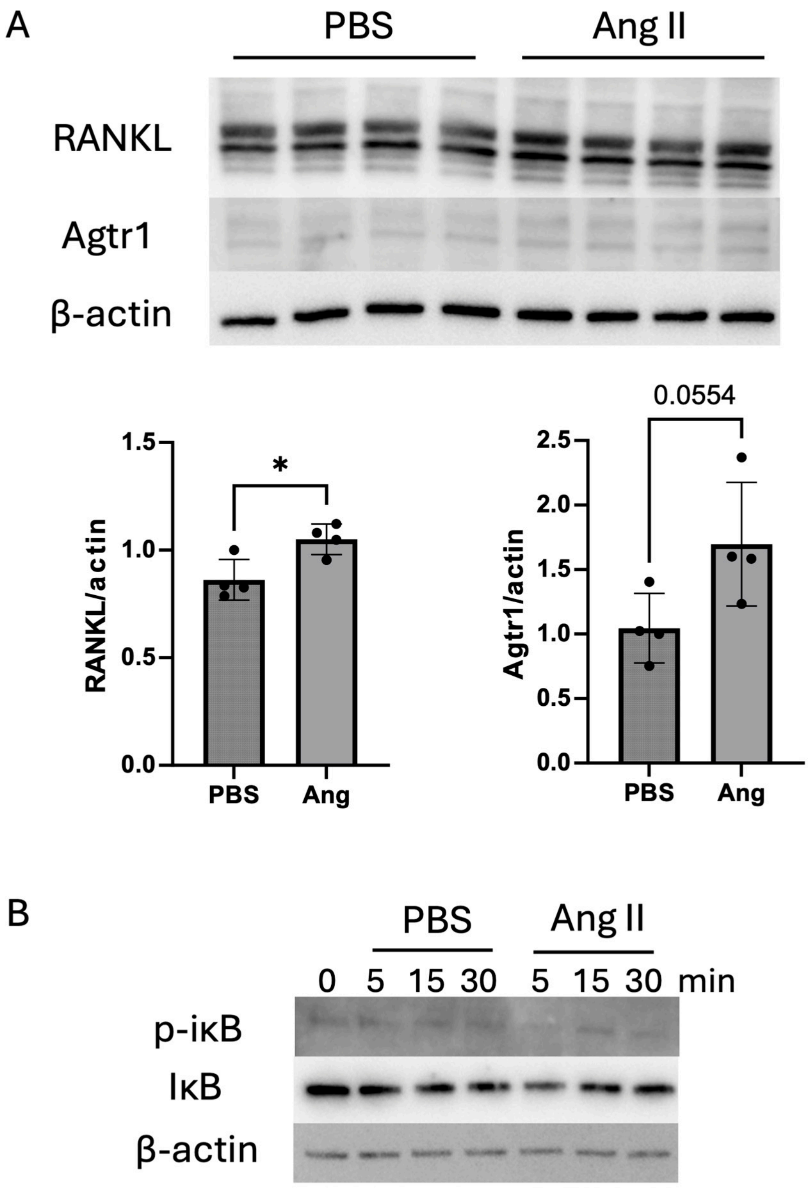

Supplementary Figure S1. A) Protein level of RANKL and Agtr1 from starved MLO-Y4 relative to β-actin. Cells were stimulated by Ang II for 24h in culture medium with 1% FBS. Unpaired t-test was used to determine statistical significance between groups ($n = 4$; * $p < 0.05$). B) NF-κB pathway activation investigated by WB ($n = 3$).
